# Correction: Improving time-resolution and sensitivity of *in situ* X-ray photoelectron spectroscopy of a powder catalyst by modulated excitation

**DOI:** 10.1039/d3sc90184j

**Published:** 2023-09-25

**Authors:** M. Roger, L. Artiglia, A. Boucly, F. Buttignol, M. Agote-Arán, J. A. van Bokhoven, O. Kröcher, D. Ferri

**Affiliations:** a Paul Scherrer Institut Forschungsstrasse 111 CH-5232 Villigen PSI Switzerland luca.artiglia@psi.ch davide.ferri@psi.ch; b École Polytechnique Fédérale de Lausanne (EPFL), Institute for Chemical Sciences and Engineering Lausanne Switzerland; c Department of Chemistry and Applied Biosciences, Institute for Chemical and Bioengineering, ETH Zurich Zurich Switzerland

## Abstract

Correction for ‘Improving time-resolution and sensitivity of *in situ* X-ray photoelectron spectroscopy of a powder catalyst by modulated excitation’ by M. Roger *et al.*, *Chem. Sci.*, 2023, **14**, 7482–7491, https://doi.org/10.1039/D3SC01274C.

The originally published version of this article contained an incorrect figure and figure caption for Fig. 5, in which the labels ‘O_2_ env.’ and ‘CO env.’ in Fig. 5b and c, respectively, were inverted. The correct Fig. 5 and corresponding caption are displayed below. Two paragraphs that refer to Fig. 5 on page 7488 in the Results and discussion section under the sub-heading ‘Identification of species on 5 wt% Pd/Al_2_O_3_’ have also been updated to replace the original text.

**Fig. 5:**
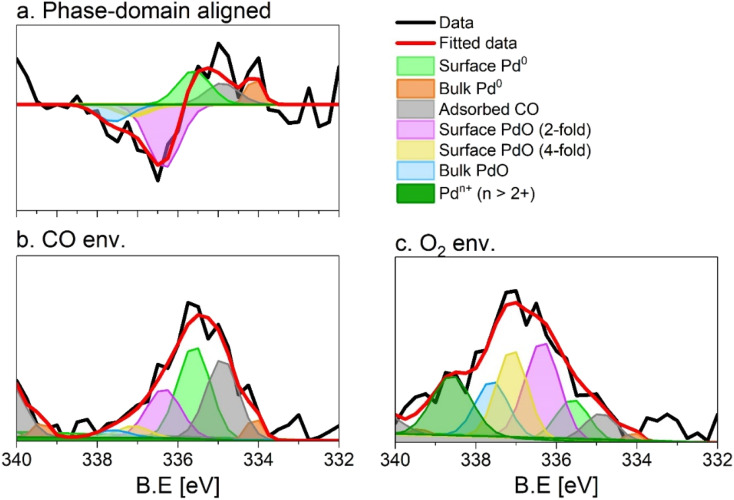
Fit of the (a) phase (0°) and (b and c) time domain spectra (*t* = 150 s), X-ray photoelectron spectra of 5 wt% Pd/Al_2_O_3_ acquired at the Pd 3d core level. (b) Averaged spectrum in reducing conditions, (c) averaged spectrum in oxidising conditions. Peaks are defined in Table 1. Only the Pd 3d_5/2_ core level is shown.

The fit of the averaged spectrum obtained at 150 s under oxidising conditions is dominated by cationic Pd species (Fig. 5c and Table 2). The contribution of Pd^0^ is low (7%), while surface Pd^0^ is absent. The fraction of Pd^0^ with adsorbed CO (11%) is not negligible and is attributed to the Pd surface poisoning by CO due to the strong Pd–CO bond. The poisoning effect of CO on Pd sites is supported by the very low levels of CO_2_ all along the O_2_ half-period in the MS data (Fig. S3), after a less pronounced sharp peak than that detected in the CO half-period. The presence of the signals of surface and bulk Pd^0^ under oxidizing conditions suggests that the thickness of the oxide layer formed is smaller than the mean escape depth of photoelectrons and thus that metallic Pd is coated by a thin skin of oxide.

Under reducing conditions, the fit of the averaged spectrum at 150 s (Fig. 5b and Table 2) shows predominantly peaks corresponding to metallic Pd species as well as Pd^0^ with adsorbed CO together with minor contributions from bulk and surface PdO but no signal of Pd^*n*+^. The poor contribution from the surface Pd^0^ (4%) is justified by the presence of adsorbed CO molecules.

The Royal Society of Chemistry apologises for these errors and any consequent inconvenience to authors and readers.

## Supplementary Material

